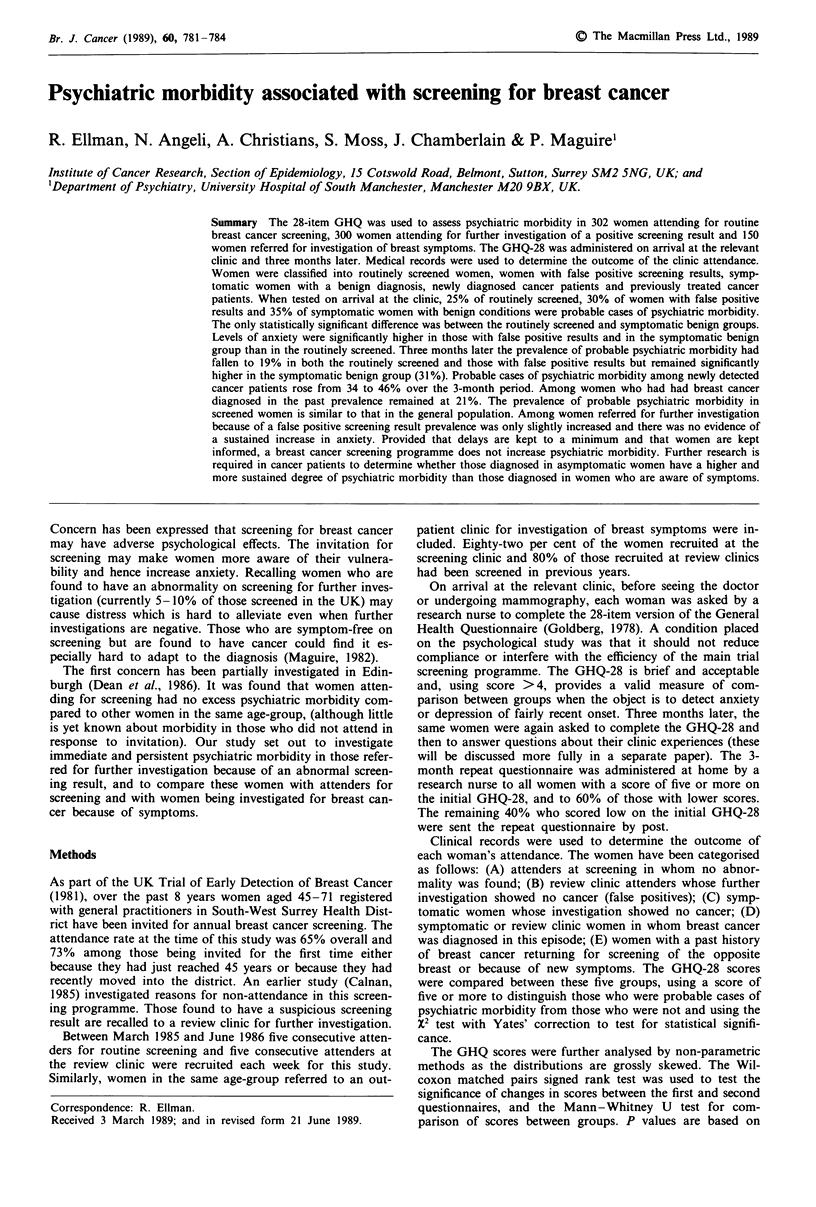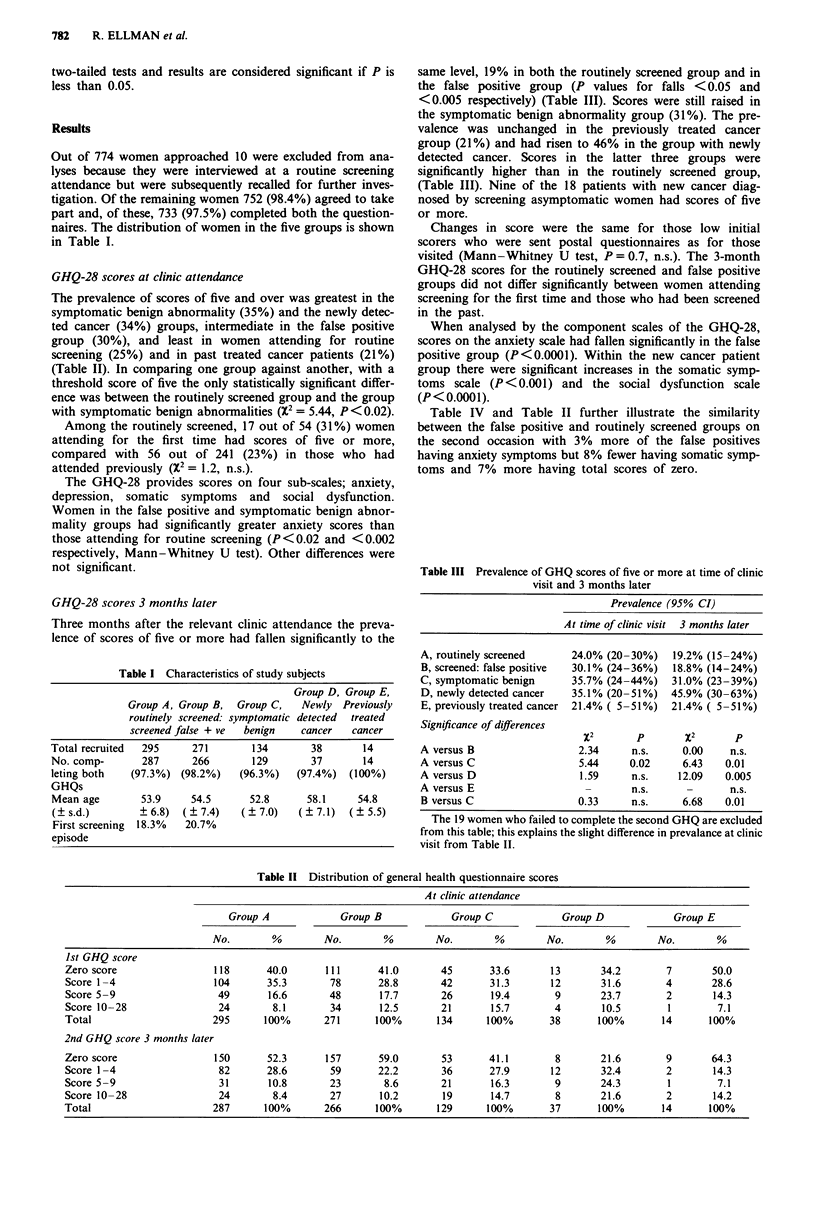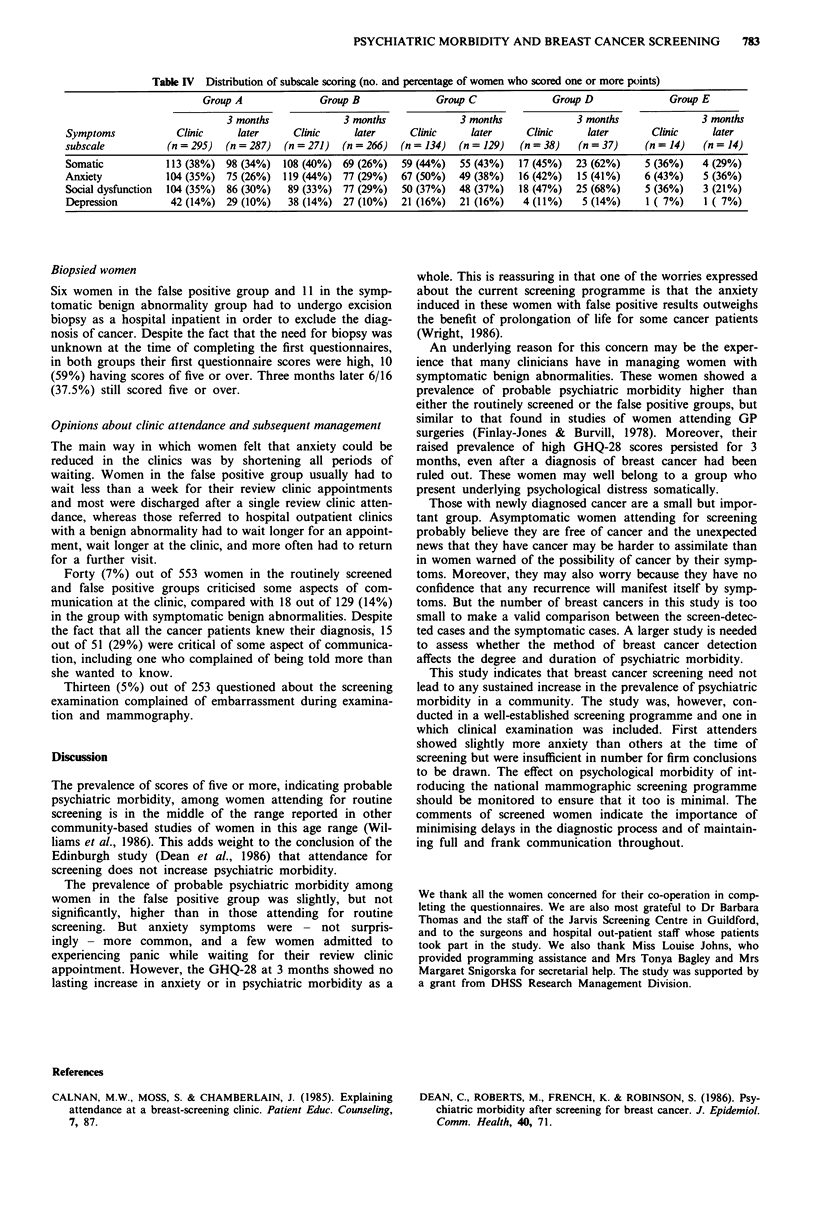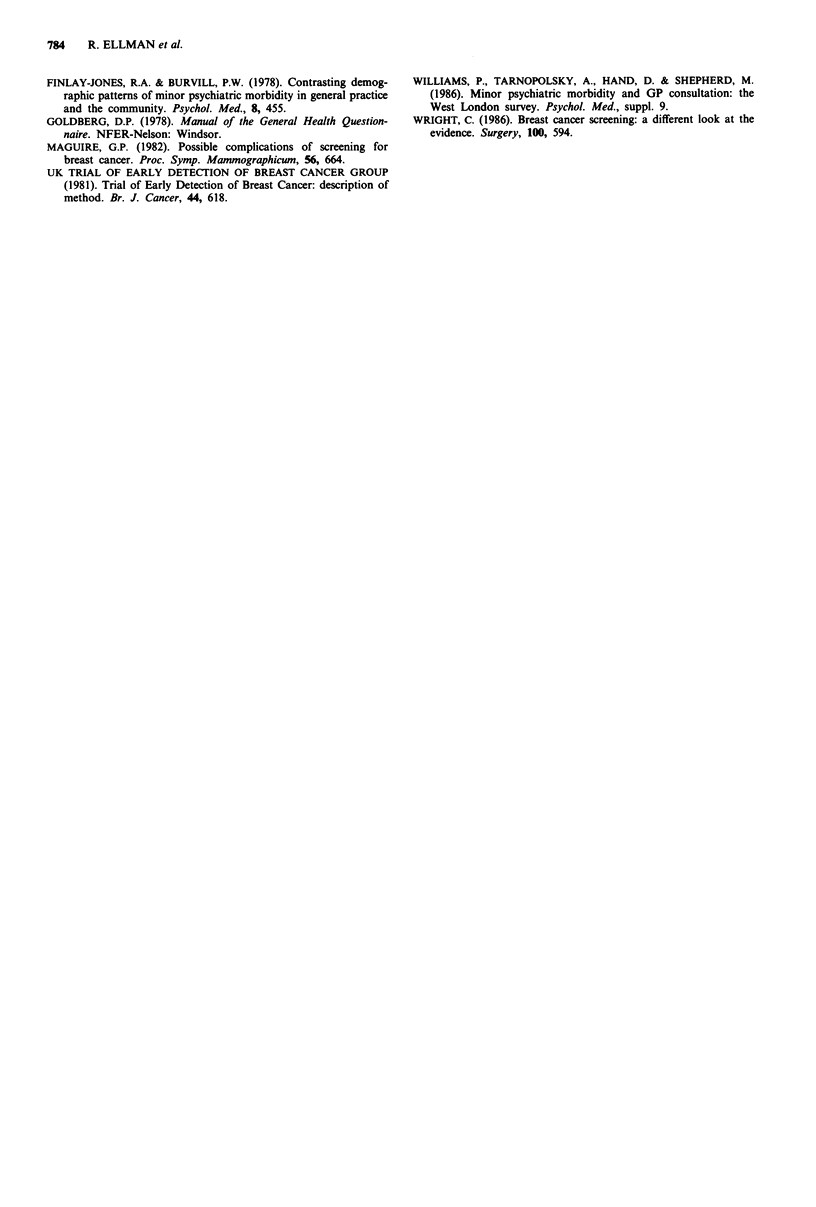# Psychiatric morbidity associated with screening for breast cancer.

**DOI:** 10.1038/bjc.1989.359

**Published:** 1989-11

**Authors:** R. Ellman, N. Angeli, A. Christians, S. Moss, J. Chamberlain, P. Maguire

**Affiliations:** Institute of Cancer Research, Section of Epidemiology, Sutton, Surrey, UK.

## Abstract

The 28-item GHQ was used to assess psychiatric morbidity in 302 women attending for routine breast cancer screening, 300 women attending for further investigation of a positive screening result and 150 women referred for investigation of breast symptoms. The GHQ-28 was administered on arrival at the relevant clinic and three months later. Medical records were used to determine the outcome of the clinic attendance. Women were classified into routinely screened women, women with false positive screening results, symptomatic women with a benign diagnosis, newly diagnosed cancer patients and previously treated cancer patients. When tested on arrival at the clinic, 25% of routinely screened, 30% of women with false positive results and 35% of symptomatic women with benign conditions were probable cases of psychiatric morbidity. The only statistically significant difference was between the routinely screened and symptomatic benign groups. Levels of anxiety were significantly higher in those with false positive results and in the symptomatic benign group than in the routinely screened. Three months later the prevalence of probable psychiatric morbidity had fallen to 19% in both the routinely screened and those with false positive results but remained significantly higher in the symptomatic benign group (31%). Probable cases of psychiatric morbidity among newly detected cancer patients rose from 34 to 46% over the 3-month period. Among women who had had breast cancer diagnosed in the past prevalence remained at 21%. The prevalence of probable psychiatric morbidity in screened women is similar to that in the general population. Among women referred for further investigation because of a false positive screening result prevalence was only slightly increased and there was no evidence of a sustained increase in anxiety. Provided that delays are kept to a minimum and that women are kept informed, a breast cancer screening programme does not increase psychiatric morbidity. Further research is required in cancer patients to determine whether those diagnosed in asymptomatic women have a higher and more sustained degree of psychiatric morbidity than those diagnosed in women who are aware of symptoms.


Br. J. Cancer (1989), 60, 781-784 © The Macmillan Press Ltd., 1989

### Psychiatric morbidity associated with screening for breast cancer

R. Ellman, N. Angeli, A. Christians, S. Moss, J. Chamberlain & P. Maguire[1]

*Institute of Cancer Research, Section of Epidemiology, 15 Cotswold Road, Belmont, Sutton, Surrey SM2 5NG, UK; and [1]Department of Psychiatry, University Hospital of South Manchester, Manchester M20 9BX, UK.*

**Summary** The 28-item GHQ was used to assess psychiatric morbidity in 302 women attending for routine breast cancer screening, 300 women attending for further investigation of a positive screening result and 150 women referred for investigation of breast symptoms. The GHQ-28 was administered on arrival at the relevant clinic and three months later. Medical records were used to determine the outcome of the clinic attendance. Women were classified into routinely screened women, women with false positive screening results, symptomatic women with a benign diagnosis, newly diagnosed cancer patients and previously treated cancer patients. When tested on arrival at the clinic, 25% of routinely screened, 30% of women with false positive results and 35% of symptomatic women with benign conditions were probable cases of psychiatric morbidity. The only statistically significant difference was between the routinely screened and symptomatic benign groups. Levels of anxiety were significantly higher in those with false positive results and in the symptomatic benign group than in the routinely screened. Three months later the prevalence of probable psychiatric morbidity had fallen to 19% in both the routinely screened and those with false positive results but remained significantly higher in the symptomatic benign group (31%). Probable cases of psychiatric morbidity among newly detected cancer patients rose from 34 to 46% over the 3-month period. Among women who had had breast cancer diagnosed in the past prevalence remained at 21%. The prevalence of probable psychiatric morbidity in screened women is similar to that in the general population. Among women referred for further investigation because of a false positive screening result prevalence was only slightly increased and there was no evidence of a sustained increase in anxiety. Provided that delays are kept to a minimum and that women are kept informed, a breast cancer screening programme does not increase psychiatric morbidity. Further research is required in cancer patients to determine whether those diagnosed in asymptomatic women have a higher and more sustained degree of psychiatric morbidity than those diagnosed in women who are aware of symptoms.

Concern has been expressed that screening for breast cancer may have adverse psychological effects. The invitation for screening may make women more aware of their vulnerability and hence increase anxiety. Recalling women who are found to have an abnormality on screening for further investigation (currently 5-10% of those screened in the UK) may cause distress which is hard to alleviate even when further investigations are negative. Those who are symptom-free on screening but are found to have cancer could find it especially hard to adapt to the diagnosis (Maguire, 1982).

The first concern has been partially investigated in Edinburgh (Dean *et al.*, 1986). It was found that women attending for screening had no excess psychiatric morbidity compared to other women in the same age-group, (although little is yet known about morbidity in those who did not attend in response to invitation). Our study set out to investigate immediate and persistent psychiatric morbidity in those referred for further investigation because of an abnormal screening result, and to compare these women with attenders for screening and with women being investigated for breast cancer because of symptoms.

### Methods

As part of the UK Trial of Early Detection of Breast Cancer (1981), over the past 8 years women aged 45-71 registered with general practitioners in South-West Surrey Health District have been invited for annual breast cancer screening. The attendance rate at the time of this study was 65% overall and 73% among those being invited for the first time either because they had just reached 45 years or because they had recently moved into the district. An earlier study (Calnan, 1985) investigated reasons for non-attendance in this screening programme. Those found to have a suspicious screening result are recalled to a review clinic for further investigation.

Between March 1985 and June 1986 five consecutive attenders for routine screening and five consecutive attenders at the review clinic were recruited each week for this study. Similarly, women in the same age-group referred to an outpatient clinic for investigation of breast symptoms were included. Eighty-two per cent of the women recruited at the screening clinic and 80% of those recruited at review clinics had been screened in previous years.

On arrival at the relevant clinic, before seeing the doctor or undergoing mammography, each woman was asked by a research nurse to complete the 28-item version of the General Health Questionnaire (Goldberg, 1978). A condition placed on the psychological study was that it should not reduce compliance or interfere with the efficiency of the main trial screening programme. The GHQ-28 is brief and acceptable and, using score $>4$, provides a valid measure of comparison between groups when the object is to detect anxiety or depression of fairly recent onset. Three months later, the same women were again asked to complete the GHQ-28 and then to answer questions about their clinic experiences (these will be discussed more fully in a separate paper). The 3-month repeat questionnaire was administered at home by a research nurse to all women with a score of five or more on the initial GHQ-28, and to 60% of those with lower scores. The remaining 40% who scored low on the initial GHQ-28 were sent the repeat questionnaire by post.

Clinical records were used to determine the outcome of each woman's attendance. The women have been categorised as follows: (A) attenders at screening in whom no abnormality was found; (B) review clinic attenders whose further investigation showed no cancer (false positives); (C) symptomatic women whose investigation showed no cancer; (D) symptomatic or review clinic women in whom breast cancer was diagnosed in this episode; (E) women with a past history of breast cancer returning for screening of the opposite breast or because of new symptoms. The GHQ-28 scores were compared between these five groups, using a score of five or more to distinguish those who were probable cases of psychiatric morbidity from those who were not and using the $\chi^2$ test with Yates' correction to test for statistical significance.

The GHQ scores were further analysed by non-parametric methods as the distributions are grossly skewed. The Wilcoxon matched pairs signed rank test was used to test the significance of changes in scores between the first and second questionnaires, and the Mann-Whitney U test for comparison of scores between groups. *P* values are based on

Correspondence: R. Ellman.
Received 3 March 1989; and in revised form 21 June 1989.

two-tailed tests and results are considered significant if $P$ is less than 0.05.

### Results

Out of 774 women approached 10 were excluded from analyses because they were interviewed at a routine screening attendance but were subsequently recalled for further investigation. Of the remaining women 752 (98.4%) agreed to take part and, of these, 733 (97.5%) completed both the questionnaires. The distribution of women in the five groups is shown in Table I.

### *GHQ-28 scores at clinic attendance*

The prevalence of scores of five and over was greatest in the symptomatic benign abnormality (35%) and the newly detected cancer (34%) groups, intermediate in the false positive group (30%), and least in women attending for routine screening (25%) and in past treated cancer patients (21%) (Table II). In comparing one group against another, with a threshold score of five the only statistically significant difference was between the routinely screened group and the group with symptomatic benign abnormalities ($\chi^2 = 5.44$, $P < 0.02$).

Among the routinely screened, 17 out of 54 (31%) women attending for the first time had scores of five or more, compared with 56 out of 241 (23%) in those who had attended previously ($\chi^2 = 1.2$, n.s.).

The GHQ-28 provides scores on four sub-scales; anxiety, depression, somatic symptoms and social dysfunction. Women in the false positive and symptomatic benign abnormality groups had significantly greater anxiety scores than those attending for routine screening ($P < 0.02$ and $< 0.002$ respectively, Mann–Whitney U test). Other differences were not significant.

### *GHQ-28 scores 3 months later*

Three months after the relevant clinic attendance the prevalence of scores of five or more had fallen significantly to the same level, 19% in both the routinely screened group and in the false positive group ($P$ values for falls $< 0.05$ and $< 0.005$ respectively) (Table III). Scores were still raised in the symptomatic benign abnormality group (31%). The prevalence was unchanged in the previously treated cancer group (21%) and had risen to 46% in the group with newly detected cancer. Scores in the latter three groups were significantly higher than in the routinely screened group, (Table III). Nine of the 18 patients with new cancer diagnosed by screening asymptomatic women had scores of five or more.

Changes in score were the same for those low initial scorers who were sent postal questionnaires as for those visited (Mann–Whitney U test, $P = 0.7$, n.s.). The 3-month GHQ-28 scores for the routinely screened and false positive groups did not differ significantly between women attending screening for the first time and those who had been screened in the past.

When analysed by the component scales of the GHQ-28, scores on the anxiety scale had fallen significantly in the false positive group ($P < 0.0001$). Within the new cancer patient group there were significant increases in the somatic symptoms scale ($P < 0.001$) and the social dysfunction scale ($P < 0.0001$).

Table IV and Table II further illustrate the similarity between the false positive and routinely screened groups on the second occasion with 3% more of the false positives having anxiety symptoms but 8% fewer having somatic symptoms and 7% more having total scores of zero.

**Table I** Characteristics of study subjects

| | *Group A, routinely screened* | *Group B, screened: false + ve* | *Group C, symptomatic benign* | *Group D, Newly detected cancer* | *Group E, Previously treated cancer* |
|---|---|---|---|---|---|
| Total recruited | 295 | 271 | 134 | 38 | 14 |
| No. completing both GHQs | 287 (97.3%) | 266 (98.2%) | 129 (96.3%) | 37 (97.4%) | 14 (100%) |
| Mean age (± s.d.) | 53.9 ± 6.8) | 54.5 ( ± 7.4) | 52.8 ( ± 7.0) | 58.1 ( ± 7.1) | 54.8 ( ± 5.5) |
| First screening episode | 18.3% | 20.7% | | | |

**Table III** Prevalence of GHQ scores of five or more at time of clinic visit and 3 months later

| | *Prevalence (95% CI)* | | | |
|---|---|---|---|---|
| | *At time of clinic visit* | | *3 months later* | |
| A, routinely screened | 24.0% (20–30%) | | 19.2% (15–24%) | |
| B, screened: false positive | 30.1% (24–36%) | | 18.8% (14–24%) | |
| C, symptomatic benign | 35.7% (24–44%) | | 31.0% (23–39%) | |
| D, newly detected cancer | 35.1% (20–51%) | | 45.9% (30–63%) | |
| E, previously treated cancer | 21.4% ( 5–51%) | | 21.4% ( 5–51%) | |
| *Significance of differences* | $\chi^2$ | $P$ | $\chi^2$ | $P$ |
| A versus B | 2.34 | n.s. | 0.00 | n.s. |
| A versus C | 5.44 | 0.02 | 6.43 | 0.01 |
| A versus D | 1.59 | n.s. | 12.09 | 0.005 |
| A versus E | – | n.s. | – | n.s. |
| B versus C | 0.33 | n.s. | 6.68 | 0.01 |

The 19 women who failed to complete the second GHQ are excluded from this table; this explains the slight difference in prevalance at clinic visit from Table II.

**Table II** Distribution of general health questionnaire scores

| | *At clinic attendance* | | | | | | | | | |
|---|---|---|---|---|---|---|---|---|---|---|
| | *Group A* | | *Group B* | | *Group C* | | *Group D* | | *Group E* | |
| | *No.* | *%* | *No.* | *%* | *No.* | *%* | *No.* | *%* | *No.* | *%* |
| *1st GHQ score* | | | | | | | | | | |
| Zero score | 118 | 40.0 | 111 | 41.0 | 45 | 33.6 | 13 | 34.2 | 7 | 50.0 |
| Score 1–4 | 104 | 35.3 | 78 | 28.8 | 42 | 31.3 | 12 | 31.6 | 4 | 28.6 |
| Score 5–9 | 49 | 16.6 | 48 | 17.7 | 26 | 19.4 | 9 | 23.7 | 2 | 14.3 |
| Score 10–28 | 24 | 8.1 | 34 | 12.5 | 21 | 15.7 | 4 | 10.5 | 1 | 7.1 |
| Total | 295 | 100% | 271 | 100% | 134 | 100% | 38 | 100% | 14 | 100% |
| *2nd GHQ score 3 months later* | | | | | | | | | | |
| Zero score | 150 | 52.3 | 157 | 59.0 | 53 | 41.1 | 8 | 21.6 | 9 | 64.3 |
| Score 1–4 | 82 | 28.6 | 59 | 22.2 | 36 | 27.9 | 12 | 32.4 | 2 | 14.3 |
| Score 5–9 | 31 | 10.8 | 23 | 8.6 | 21 | 16.3 | 9 | 24.3 | 1 | 7.1 |
| Score 10–28 | 24 | 8.4 | 27 | 10.2 | 19 | 14.7 | 8 | 21.6 | 2 | 14.2 |
| Total | 287 | 100% | 266 | 100% | 129 | 100% | 37 | 100% | 14 | 100% |

**Table IV** Distribution of subscale scoring (no. and percentage of women who scored one or more points)

| | Group A | | Group B | | Group C | | Group D | | Group E | |
|---|---|---|---|---|---|---|---|---|---|---|
| *Symptoms subscale* | *Clinic (n = 295)* | *3 months later (n = 287)* | *Clinic (n = 271)* | *3 months later (n = 266)* | *Clinic (n = 134)* | *3 months later (n = 129)* | *Clinic (n = 38)* | *3 months later (n = 37)* | *Clinic (n = 14)* | *3 months later (n = 14)* |
| Somatic | 113 (38%) | 98 (34%) | 108 (40%) | 69 (26%) | 59 (44%) | 55 (43%) | 17 (45%) | 23 (62%) | 5 (36%) | 4 (29%) |
| Anxiety | 104 (35%) | 75 (26%) | 119 (44%) | 77 (29%) | 67 (50%) | 49 (38%) | 16 (42%) | 15 (41%) | 6 (43%) | 5 (36%) |
| Social dysfunction | 104 (35%) | 86 (30%) | 89 (33%) | 77 (29%) | 50 (37%) | 48 (37%) | 18 (47%) | 25 (68%) | 5 (36%) | 3 (21%) |
| Depression | 42 (14%) | 29 (10%) | 38 (14%) | 27 (10%) | 21 (16%) | 21 (16%) | 4 (11%) | 5 (14%) | 1 ( 7%) | 1 ( 7%) |

*Biopsied women*

Six women in the false positive group and 11 in the symptomatic benign abnormality group had to undergo excision biopsy as a hospital inpatient in order to exclude the diagnosis of cancer. Despite the fact that the need for biopsy was unknown at the time of completing the first questionnaires, in both groups their first questionnaire scores were high, 10 (59%) having scores of five or over. Three months later 6/16 (37.5%) still scored five or over.

*Opinions about clinic attendance and subsequent management*

The main way in which women felt that anxiety could be reduced in the clinics was by shortening all periods of waiting. Women in the false positive group usually had to wait less than a week for their review clinic appointments and most were discharged after a single review clinic attendance, whereas those referred to hospital outpatient clinics with a benign abnormality had to wait longer for an appointment, wait longer at the clinic, and more often had to return for a further visit.

Forty (7%) out of 553 women in the routinely screened and false positive groups criticised some aspects of communication at the clinic, compared with 18 out of 129 (14%) in the group with symptomatic benign abnormalities. Despite the fact that all the cancer patients knew their diagnosis, 15 out of 51 (29%) were critical of some aspect of communication, including one who complained of being told more than she wanted to know.

Thirteen (5%) out of 253 questioned about the screening examination complained of embarrassment during examination and mammography.

### Discussion

The prevalence of scores of five or more, indicating probable psychiatric morbidity, among women attending for routine screening is in the middle of the range reported in other community-based studies of women in this age range (Williams *et al.*, 1986). This adds weight to the conclusion of the Edinburgh study (Dean *et al.*, 1986) that attendance for screening does not increase psychiatric morbidity.

The prevalence of probable psychiatric morbidity among women in the false positive group was slightly, but not significantly, higher than in those attending for routine screening. But anxiety symptoms were – not surprisingly – more common, and a few women admitted to experiencing panic while waiting for their review clinic appointment. However, the GHQ-28 at 3 months showed no lasting increase in anxiety or in psychiatric morbidity as a whole. This is reassuring in that one of the worries expressed about the current screening programme is that the anxiety induced in these women with false positive results outweighs the benefit of prolongation of life for some cancer patients (Wright, 1986).

An underlying reason for this concern may be the experience that many clinicians have in managing women with symptomatic benign abnormalities. These women showed a prevalence of probable psychiatric morbidity higher than either the routinely screened or the false positive groups, but similar to that found in studies of women attending GP surgeries (Finlay-Jones & Burvill, 1978). Moreover, their raised prevalence of high GHQ-28 scores persisted for 3 months, even after a diagnosis of breast cancer had been ruled out. These women may well belong to a group who present underlying psychological distress somatically.

Those with newly diagnosed cancer are a small but important group. Asymptomatic women attending for screening probably believe they are free of cancer and the unexpected news that they have cancer may be harder to assimilate than in women warned of the possibility of cancer by their symptoms. Moreover, they may also worry because they have no confidence that any recurrence will manifest itself by symptoms. But the number of breast cancers in this study is too small to make a valid comparison between the screen-detected cases and the symptomatic cases. A larger study is needed to assess whether the method of breast cancer detection affects the degree and duration of psychiatric morbidity.

This study indicates that breast cancer screening need not lead to any sustained increase in the prevalence of psychiatric morbidity in a community. The study was, however, conducted in a well-established screening programme and one in which clinical examination was included. First attenders showed slightly more anxiety than others at the time of screening but were insufficient in number for firm conclusions to be drawn. The effect on psychological morbidity of introducing the national mammographic screening programme should be monitored to ensure that it too is minimal. The comments of screened women indicate the importance of minimising delays in the diagnostic process and of maintaining full and frank communication throughout.

We thank all the women concerned for their co-operation in completing the questionnaires. We are also most grateful to Dr Barbara Thomas and the staff of the Jarvis Screening Centre in Guildford, and to the surgeons and hospital out-patient staff whose patients took part in the study. We also thank Miss Louise Johns, who provided programming assistance and Mrs Tonya Bagley and Mrs Margaret Snigorska for secretarial help. The study was supported by a grant from DHSS Research Management Division.

### References

CALNAN, M.W., MOSS, S. & CHAMBERLAIN, J. (1985). Explaining attendance at a breast-screening clinic. *Patient Educ. Counseling*, **7**, 87.

DEAN, C., ROBERTS, M., FRENCH, K. & ROBINSON, S. (1986). Psychiatric morbidity after screening for breast cancer. *J. Epidemiol. Comm. Health*, **40**, 71.

FINLAY-JONES, R.A. & BURVILL, P.W. (1978). Contrasting demographic patterns of minor psychiatric morbidity in general practice and the community. *Psychol. Med.*, **8**, 455.

GOLDBERG, D.P. (1978). *Manual of the General Health Questionnaire*. NFER-Nelson: Windsor.

MAGUIRE, G.P. (1982). Possible complications of screening for breast cancer. *Proc. Symp. Mammographicum*, **56**, 664.

UK TRIAL OF EARLY DETECTION OF BREAST CANCER GROUP (1981). Trial of Early Detection of Breast Cancer: description of method. *Br. J. Cancer*, **44**, 618.

WILLIAMS, P., TARNOPOLSKY, A., HAND, D. & SHEPHERD, M. (1986). Minor psychiatric morbidity and GP consultation: the West London survey. *Psychol. Med.*, suppl. 9.

WRIGHT, C. (1986). Breast cancer screening: a different look at the evidence. *Surgery*, **100**, 594.